# Topographic relationship between root apex of mesially and horizontally impacted mandibular third molar and lingual plate: cross-sectional analysis using CBCT

**DOI:** 10.1038/srep39268

**Published:** 2016-12-19

**Authors:** Dongmiao Wang, Xiaotong He, Yanling Wang, Guangchao Zhou, Chao Sun, Lianfeng Yang, Jianling Bai, Jun Gao, Yunong Wu, Jie Cheng

**Affiliations:** 1 Department of Oral and Maxillofacial Surgery, College of Stomatology, Nanjing Medical University, Nanjing 210029, China; 2 Jiangsu Key Laboratory of Oral Disease, Nanjing Medical University, Nanjing 210029, China; 3 Department of oral prosthodontics, College of Stomatology, Nanjing Medical University, Nanjing 210029, China; 4 Department of Oral and Maxillofacial Radiology, College of Stomatology, Nanjing Medical University, Nanjing 210029, China; 5 Department of Biomedical Statistics, College of Public Health, Nanjing Medical University, Nanjing 210029, China

## Abstract

The present study was aimed to determine the topographic relationship between root apex of the mesially and horizontally impacted mandibular third molar and lingual plate of mandible. The original cone beam computed tomography (CBCT) data of 364 teeth from 223 patients were retrospectively collected and analyzed. The topographic relationship between root apex and lingual plate on cross-sectional CBCT images was classified as non-contact (99), contact (145) and perforation (120). The cross-sectional morphology of lingual plate at the level of root apex was defined as parallel (28), undercut (38), slanted (29) and round (4). The distribution of topographic relationship between root apex and lingual plate significantly associated with gender, impaction depth, root number and lingual plate morphology. Moreover, the average bone thickness of lingual cortex and distance between root apex and the outer surface of lingual plate were 1.02 and 1.39 mm, respectively. Furthermore, multivariate regression analyses identified impaction depth and lingual plate morphology as the risk factors for the contact and perforation subtypes between root apex and lingual plate. Collectively, our findings reveal the topographic proximity of root apex of impacted mandibular third molar to the lingual plate, which might be associated with intraoperative and postoperative complications during tooth extraction.

The mandibular third molars are the most frequently impacted teeth largely due to the lack of adequate space in the lower jaw or the barriers in their eruption trajectory. These impacted teeth usually result in diverse types of pathological conditions including reiterative pericoronitis, swelling, odontogenic cysts or tumors, bone loss as well as root resorption of the adjacent teeth, thus inevitably leading to impaired oral functions and discomfort^1^,^2^,^3^. The extraction of impacted mandibular third molar is one of the most common surgical procedures in the dental clinic and outpatient department of oral and maxillofacial surgery. Similar as other treatments, various types of intraoperative and postoperative complications might occur including infection, limited mouth opening, inferior alveolar nerve and lingual nerve damage, *et al*.^4^,^5^,^6^,^7^. Among them, fracture of lingual plate and displacement of roots or root fragments into adjacent fascial spaces have been reported and not rarely observed in the clinical practice, although the incidences are unknown^8^,^9^,^10^. Such events might result in bleeding, infection of submandibular and pterygomandibular spaces and lingual nerve injury^6^,^11^,^12^. Therefore, how to prevent the occurrence of these unwanted complications and identify the relevant risk factors remains largely underexplored yet.

Previous studies have proposed that the risk factors for lingual plate fracture, root fragment displacement as well as lingual nerve damage are associated with thin lingual cortical plate or its fenestration, poor surgical skills and depth and angulation of impaction, *et al*.^6^,^9^,^13^. Currently, identifying the relevant risk factors associated with complications during and after the impacted third molar surgery primarily depends on presurgical radiographic examinations. For example, panoramic radiography is a routine and widely used approach for preoperative risk assessment in third molar surgery^14^,^15^. However, panoramic radiography can’t definitely identify the precise topographic positions of the tooth and its root, and clearly determine the integrity of the lingual plate directly due to their two-dimensional nature, unequal magnification as well as possible image distortions^16^,^17^. Darkening in the roots of impacted lower third molar was considered to reflect the lingual cortical thinning or perforation which still remains controversial^18^,^19^. The advent of cone beam computed tomography (CBCT) in dentistry enable clinicians to visualize the dental and maxillofacial structures more precisely and clearly from both multiple planes and 3-dimentional views^20^,^21^,^22^,^23^. It has multiple advantages such as high spatial resolution, low radiation dosage as well as multiplanar images free of overlapping as compared to other dental radiography or conventional CT^20^,^21^.

Lingual plate morphology and bone thickness in mandible have been increasingly recognized as key factors to avoid unpleasant complications during implant placement and extraction of mandibular impacted molars^13^,^24^,^25^,^26^,^27^,^28^. Previous studies have shown diverse types of the morphology of lingual plate (convergent, parallel, undercut) at the post-mandibular region on cross-sectional CBCT images^24^. Emes *et al*. measured the distance between the root apex of third molars and lingual cortical plate with average 1.03 mm by CBCT. Their data indicated that root apex of impacted third molar can be very close to the lingual plate and sometimes even protruded into soft tissue of mouth floor in 25% cases^26^. Additionally, Tolstunov and his colleagues reported that fenestration in the lingual plate identified by CBCT scan was more commonly observed than originally expected at the third molar apex region and such fenestration of lingual bone was significantly associated with the angulation of the impacted third molar^29^. Moreover, from CBCT data of 110 deeply impacted mandibular third molars, the authors reported that 87.3% teeth were classified as lingual position according to their classification based on the deduction values of buccal-lingual alveolar bone thickness^13^. Collectively, these abovementioned findings support the notion that these anatomic factors might contribute to fracture of lingual plate, root displacement as well as lingual nerve injury after extraction of impacted mandibular third molars. However, comprehensive analyses of topographic relationship between the roots of mesially and horizontally impacted mandibular third molars and mandibular lingual plate are still lacking thus far.

The aim of this study was to measure the thickness of the lingual plate at the level of root apex of impacted mandibular third molar and determine the topographic relationship between the root apex and lingual plate using CBCT images. We also sought to identify the potential associated factors with the perforation of lingual plate at this region.

## Results

**Figure 1:**
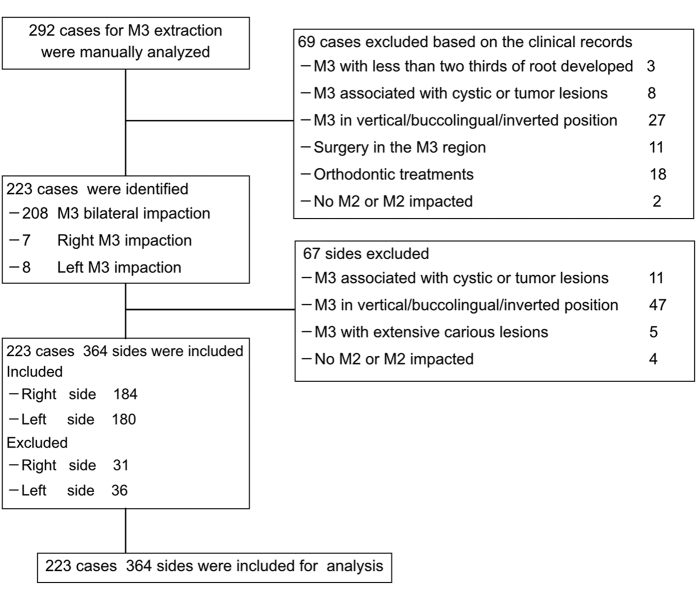
Patients screen and inclusion protocol.

Through data screen and analyses in our clinical patients registry as described in Fig. 1, we identified 223 patients satisfying the inclusion criteria from a total number of 292 patients who received impacted lower third molar extraction during the last four years (Jan.2012–Dec.2015). As listed in Table 1, the mean age was 30.42 ± 9.71 years (range: 18–69 years), with 117 (52.47%) males and 106 (47.53%) females. In addition, based on the depth of impaction, these impacted mandibular third molars were categorized into 99 as Class A, 213 as Class B, and 52 as Class C impaction, respectively. Furthermore, unilateral impaction of the mandibular third molar was present in 15 patients (7 at the right, 8 at the left), and the others were presented with bilateral impactions. Among them, 67 sides (31 at the right, 36 at the left) were further excluded according to the exclusion criteria. Finally, the original CBCT data of these 223 patients involving 364 impacted mandibular third molars (184 at the right, 180 at the left) were retrieved and further analyzed here.

To verify the reliability and reproducibility of our research methods based on CBCT images, our initial studies concerning the classifications of lingual plate morphology and impaction depth of mandibular third molar revealed good inter-observer reliability (κ = 0.9246, *P* = 0.0015) as estimated by the κ-test analyses. No statistical significance between two observers with regard to the data about the thickness of lingual plate as well as the distance between root apex and lingual plate in randomly selected cases was observed (*P* > 0.05, Student-*t* test, Supplementary Table 1 and data not shown). Moreover, there was no significant difference between the data from one observer who performed the same measurements in the same cases (*P* > 0.05, Student-*t* test, Supplementary Table 2 and data not shown). Collectively, these findings clearly suggest that our research protocols for the measurements and categorizations are reproducible and reliable as evidenced by good intra- and inter-observer reliability.

All included third molars were categorized as 99 (27.20%) in non-contact group, 145 (39.83%) in contact group and 120 (32.97%) in perforation group based on the spatial relationship between root apex and lingual plate. In addition, the morphology of lingual plate at the level of root apex on the cross-sectional CBCT images were further classified into 28 parallel, 38 undercut, 29 slanted and 4 round subgroups. As detailed in Table 2, there were significant associations found between the root-lingual plate topography and gender, impaction depth, root numbers as well as lingual plate shape, respectively (*P*-values less than 0.05, Chi-square test). In addition, no significant association was detected between the lingual positions of the impacted third molar roots and patients’ age (*P* > 0.05, Chi-square test).

The mean thickness of lingual cortical plate at the level of impacted third molar apex is 1.02 mm. As shown in Table 3, significant difference of the thickness among different types of spatial relationship between root and lingual plate (non-contact vs contact type) was found with *P*-value less than 0.0001 (Student-*t* test). Additionally, the lingual plate in non-contact group was significantly thicker than that in other groups. Furthermore, our data indicate that the distance between the root apex of impacted third molar and the outer border of lingual plate as measured on cross-sectional CBCT image was 1.39 ± 1.32 mm. As displayed in Table 4, there was significant difference of root apex-lingual plate distance between two types of root-lingual spatial relationship (non-contact vs contact type) with *P*-value less than 0.0001 (Student-t test). As expected, such distance was the longest in non-contact subgroup.

To strengthen the clinical significance of these abovementioned radiographic findings, we next performed multivariate analyses via logic regression assay to identify the potential risk factors for diverse types of spatial relationship between root apex of mesially/horizontally impacted mandibular third molar and lingual plate of mandible. Several parameters such as age, gender, impaction depth, number of root as well as morphology of lingual plate were included. As shown in Table 5, when contact subgroup was in comparison to non-contact subgroup, depth of impaction (Type B and C) appeared to be negatively associated with the occurrence of direct contact between root apex and lingual plate, while the slanted shape of lingual plate was positively associated with the possibility of the contact with odds ratio (OR) 2.44 (*P* = 0.0244). Moreover, when perforation subgroup was compared with non-contact subgroup, age between 26–35 and undercut/slanted shape of lingual plate appeared to be significantly associated with root perforation beyond lingual plate (OR = 2.16, *P* = 0.0274; OR = 4.45, *P* = 0.0.0010; OR = 2.71, *P* = 0.0374; respectively). Meanwhile, female and impaction depth (Type C) seemed to be negatively associated with such lingual plate perforation (OR = 0.47, *P* = 0.0457; OR = 0.14, *P* = 0.0.0004; respectively).

## Discussion

The anatomic morphology of the posterior mandible has increasingly attracted the attentions and interests in the dental clinic largely due to its importance and contributions to potential postsurgical complications following the third molar surgery and dental implant surgery^24^,^26^,^27^,^30^. A line of evidence has suggested that the thin and shape of lingual cortical plate as well as perforation or fenestration in lingual plate are associated with accidental displacement of third molars or root fragments, secondary infection of submandibular space and lingual nerve injury following surgical extraction of impacted third molars^8^,^10^,^26^,^31^. Therefore, comprehensive understanding and analyses of the anatomy in lingual plate of mandible and its spatial relationship with impacted third molars before surgery might be beneficial to prevent the complications and communicate with patients regarding the surgical risks, especially for those cases with high risks. Here we focus on the spatial proximity between root apex of mesially and horizontally impacted third molar by analyzing the cross-sectional CBCT images. Our findings reveal that the root apex of mandibular third molar with mesial and horizontal impactions have close spatial relationship with lingual plate, sometimes direct contact or perforation beyond the outer border of lingual plate.

Previously, the dental panoramic radiography was commonly exploited to observe the anatomic structures and detect the pathological lesions in both maxilla and mandible. Notably, it is also routinely used to observe the depth and angulation of impacted mandibular third molar and discern its topographic relationship with inferior alveolar canal^15^,^32^. However, this two-dimensional technique has inherent weakness including more artifacts, image distortion and overlapping, which precludes it to accurately evaluate the local anatomy of mandible in detail. Three-dimensional imaging techniques such as CT and CBCT have proved to be advantageous relative to the two-dimensional radiography in presurgical evaluations before third molar surgery^21^. Moreover, high spatial resolution, better-quality view of dental structures, few artifacts and relatively low radiographic dosage enable CBCT as a preferred imaging technique to detect both normal and pathological conditions in the dentomaxillofacial region^20^. In the present study, we used CBCT data and 3-dimentional software to observe the anatomic location of the root of impacted third molar and its spatial relationship with mandibular lingual plate. Our radiographic findings such as distance measurements and tooth categorizations were well consistent between observers and also had good intra-observer reliability, thus suggesting the reliability and reproducibility of our methods using CBCT images.

The distribution of spatial relationship between root apex of impacted third molar and lingual plate varied among diverse previous studies^26^,^27^,^29^. Emes and his colleagues reported that only four root apexes of impacted third molars were identified on CBCT images which perforated beyond the lingual plate and directly contacted with lingual soft tissue (Type C) in 32 teeth (31 patients)^26^. However, Tolstunov L, *et al*. recently reported the incidence of root perforation in the lingual cortex was much higher (65.5%) as detected by CBCT in 149 patients with 200 partially impacted mandibular third molars^29^. Here, we included 364 mesially and horizontally impacted mandibular third molars and found that 32.97% (120/364) root apexes perforated beyond the outer border of the lingual cortex and 40.28% (145/360) were direct contact with lingual cortical bone. Such incidences were much lower as compared with Tolstunov’s data^29^, but markedly higher in relative to Emes’s findings^26^. We reasoned that this discrepancy might associate with different sample size, bias of patient selection, varied criteria of patient inclusion and categorification methods. Moreover, the average distance between root apex in the most lingual position and lingual cortical plate in the present study was in agreement with in Emes’s report (1.03 mm)^26^. The average thickness of lingual plate was 1.02 mm which seems larger than Momin’s data (0.68 mm)^27^ and fewer than Ge’s data (1.54 mm)^13^. This difference might be attributed to different measurement methods as well as case selection. Together, these findings suggest that the root apex of impacted third molar is spatially close to lingual plate of mandible, which probably represents an anatomic factor associated with the vulnerability of accidental displacement of root fragments and lingual plate fracture during impacted third molar extraction.

Noticeably, our data further revealed that the topographic relationship between root apex and lingual cortical plate is significantly associated with gender, impaction depth, root numbers and lingual plate shape. Although Momin MA reported no gender-specific difference in anatomic risk of lingual plate fracture^27^, our data is partially supported by Sathapana’s findings that considerable difference of lingual bone thickness is observed in the retromolar region in men and women, and cortical bone increases more with age in women than in men^33^. However, we can’t rule out the other possibility to account for this gender difference, for example sample size, selection bias as well as measurement difference. In addition, deep impaction seems significantly associated with decreased incidence and risk of lingual plate perforation. We assume that with impaction depth increase, the root of third mandibular molar become progressively narrowed and might have few possibility to contact or even perforate the lingual cortex. Of course, this hypothesis is needed to be further confirmed radiographically and clinically. Moreover, multiple roots of the impacted third molars appears to have higher incidence of lingual plate contact and perforation as compared to those with single root, although this is not supported by our multivariate regression assay.

Accumulating evidence has revealed that lingual concavity or undercut is common in the posterior mandible which associates with high risks for complications after third molar extraction as well as dental implant placement^25^,^26^,^34^. The prevalence and degree of lingual concavity in mandible as well as its morphological classification have been well documented^27^,^28^,^30^. Our data indicated that the undercut shape was the most common morphology of lingual plate at the third molar region followed by slanted, parallel and round shape. This is consistent with Lin MH’s^34^, Huang RY’s^25^, Chan HL’s reports^30^, but in contrast to Momin MA’s^27^ and Watanabe’s findings^28^. This heterogeneity of prevalence might be attributed to different classifications employed, ethnicity, diverse locations of interest as well as the presence/absence of teeth. Moreover, our findings revealed that diverse shape of lingual plate was significantly associated with the lingual position of root apex of impacted third molars and its spatial relationship with lingual plate. This was further supported by the regression analyses in which undercut and slanted shapes were identified as risk factors for contact/perforation between root apex and lingual plate. Together, these data support that presurgical knowledge of lingual plate shape in the impacted third molar region will remind the dentists or surgeons to evaluate the risks of lingual plate fracture and root fragment displacement during tooth extraction.

There are several advantages and limitations concerning our findings. The patients included here were strictly filtered and screened according to our inclusion/exclusion criteria to reduce sample heterogeneity as much as possible. Here only these mesially and horizontally impacted mandibular third molars were enrolled largely due to their major prevalence in population^35^ and intimate topographic relationship between the roots and lingual plate of mandible^29^. To the best of our knowledge, our sample size might be the largest one to evaluate the spatial relationship between impacted third molars and lingual plate by cross-sectional CBCT images. However, more data from a large amount of patients or from diverse races is still needed to further validate our findings. Moreover, the present study is a retrospective study with potential bias of sample selection. In the present study, we determined the anatomic proximity of impacted third molar root apex to lingual plate and measured the distance between root apex and lingual plate as well as thickness of lingual plate at the level of root apex on the cross-sectional CBCT images. However, comprehensive analyses of the bone morphology and thickness as well as anatomic proximity between root and lingual plate at diverse levels of tooth such as the apical half and cervical levels might offer more information to the clinicians to fully assess the risks before extraction. Furthermore, studies are warranted to assess the bone around the third molar roots, the angle/width/depth of lingual concavity, and topographic relationship between the undercut point and root tip. To reinforce the clinical significance of our findings, the clinical data of patients such as the occurrence of intraoperative lingual plate fracture, root fragment displacement, secondary infection as well as lingual nerve injury will provided further evidence to support our radiographic findings.

In conclusion, our data reveal that the root apex of mesially and horizontally impacted third molars are topographically closed to the lingual plate of mandible which is not rare as previously expected and can be clearly identified via CBCT scan and image analyses. These anatomic factor might contribute to lingual plate fracture, displacement of roots or root fragments and lingual nerve surgery during third molar surgery and associate with postoperative complications. When the topographic proximity between root apex of impacted third molars and lingual plate exists, the clinicians might communicate well with the patients regarding the potential surgical risks and stay alert during tooth extraction.

## Material and Methods

### Patients screen and inclusion

This was a retrospective cohort study of patients who were treated at the Department of Oral and Maxillofacial Surgery, Affiliated Hospital of Stomatology, Nanjing Medical University, for surgical extraction of the impacted mandibular third molars between January 2012 and December 2015. The research protocol (2016-156) was reviewed and approved by the Ethics and Research Committee of Nanjing Medical University. Written informed consent was obtained from all enrolled patients before surgery. The methods and protocols in the whole study were performed in accordance with the tenets of the Declaration of Helsinki for research involving human subjects as well as our institutional guidelines.

Given the mesioangular and horizontal impaction as the most prevalent type of mandibular third molar impaction and intimate spatial relationship between its root and lingual plate^29^,^35^, here the adult patients (age over 18 years old) with mesially or horizontally impacted mandibular third molars were initially included. The category of impaction of each tooth was identified with presurgical panoramic radiography and/or CBCT scans, and recorded based on Winter’s classifications as described before^3^,^28^. Furthermore, the patient exclusion criteria were listed as follows: 1) the impacted mandibular third molars associated with cystic or tumor lesions, 2) the teeth with less than two thirds of root developed, 3) the teeth showing extensive carious lesion, 4) the adjacent second molars which were extracted or impacted simultaneously, 5) oral surgical procedures involved in the mandibular third molar region, 6) historical or ongoing orthodontic treatments, 7) the poor quality of CBCT images which jeopardized unambiguous view of local anatomy and structures. The detailed screen and inclusion/exclusion of patients were described in Fig. 1.

### CBCT image acquisition and analysis

**Figure 2:**
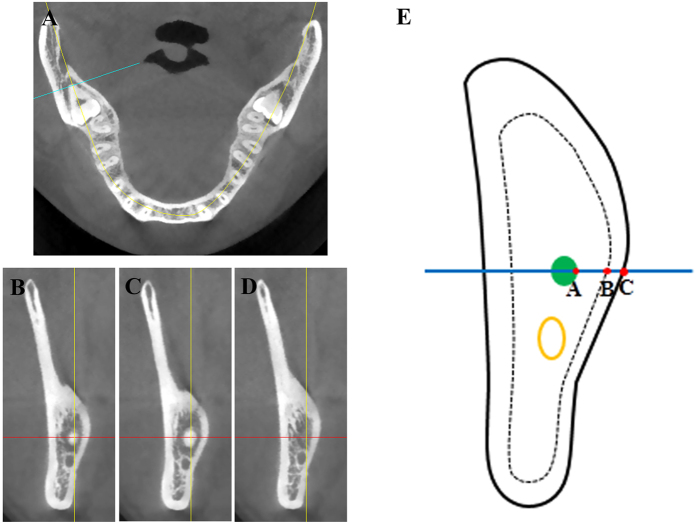
Selection of cross-sectional CBCT images when root apex of impacted mandibular third molar was located at its mostly distal slice and subsequent relevant measurements. (**A**) The root apex of impacted mandibular third molar which was localized most distally was initially identified on the axial CBCT image. (**B**–**D**) Starting from the (**A**) image, the CBCT slice was scrolled distally or mesially. When the root tip was enlarged (**C**) or disappeared (**D**) on the next slices after the image was adjusted mesially or distally (0.1 mm). the present image (**B**) on the cross-sectional plane was further selected for various measurements and spatial analyses. (**E**) Schematic illustration of the measurements. Point A was identified as the most lingual site of root apex. A horizontal line through point A was automatically generated which was intersected with the inner (Point B) and outer surface (Point C) of lingual plate, respectively. The measurements such as thickness of lingual plate (**B**–**C**) and distance between root apex and the outer surface of lingual plate (**A**–**C**) were performed using the digital ruler provided by the software Simplant.

Although the presurgical CBCT scan is not a prerequisite or routinely prescribed for impacted third molar extraction, patients included here had CBCT scan after the initial panoramic radiography suggested the proximity between the root of impacted third molar and mandibular canal, surgical difficulties as well as the risk of nerve injury. Preoperative CBCT images of oral and maxillofacial region including the mandible were acquired with a CBCT scanner (New Tom VG, Verona, Italy) at medium volume and high resolution. The operating parameters were 7.3 mA and 110 kV with a 0.5 mm fixed focal spot and 18 × 16 cm field of view. The qualified CBCT images were analyzed with the Simplant program (version 16.0, DENTSPLY International) from axial, sagittal and cross-sectional planes by two trained observers with high expertise (Dr. Dongmiao Wang and Dr. Xiaotong He) as shown in Fig. 2. When different ideas concerning the radiographic findings on CBCT images occur, they should discuss with another independent observer (Dr.Yanling Wang) and then obtain the final consensus. In brief, the root apex of impacted third molar was initially identified on the axial plane. If the tooth was multi-rooted, the root at the most lingual position was considered and selected for study. The section in which the apex was the most distal was further selected (Fig. 2A) and recognized on the cross-sectional plane. Then we scrolled the image distally and mesially and determined the root apex on the cross-sectional plane. When the root tip was enlarged (Fig. 2C) or disappeared (Fig. 2D) on the next slices after the image was adjusted mesially or distally (0.1 mm). the present image (Fig. 2B) on the cross-sectional plane was further selected for various measurements and spatial analyses. As schematically depicted in Fig. 2E, point A was identified as the most lingual site of root apex. A horizontal line through point A was generated which was intersected with the inner (Point B) and outer surface (Point C) of lingual plate, respectively. The measurements such as thickness of lingual plate (B-C) and distance between the root apex and the outer surface of lingual plate (A-C) were performed accordingly using the digital ruler provided by the software. Each measurement was repeated three times and the data were further averaged and recorded.

**Figure 3:**
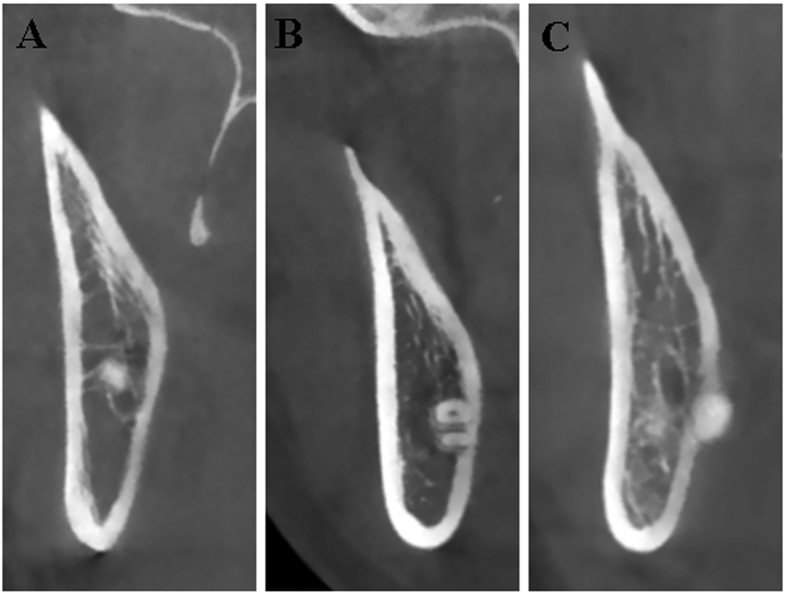
Topographic relationship between root apex of impacted mandibular third molar and lingual plate. (**A**) Type A (non-contact), there is space between the root surface and the inner boarder of lingual plate; (**B**) Type B (contact), the root apex contacts with lingual plate directly; (**C**) Type C (perforation), the root apex perforates beyond the outer boarder of lingual plate.

**Figure 4:**
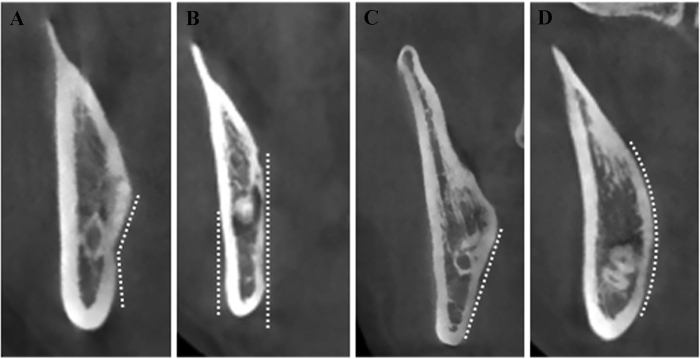
Morphology of lingual plate at the level of root apex identified on cross-sectional CBCT image. (**A**) Type U, undercut on the lingual side; (**B**) Type P, parallel to the buccal plate; (**C**) Type S, slanted with buccolingual width reduced on the lingual side; (**D**) Type R, round shape on the lingual side.

Similarly as Emes Y, *et al*. reported before^26^, the lingual positions of impacted third molar roots were categorized into three groups on the cross-sectional plane as shown in Fig. 3. Type A (non-contact, Fig. 3A) is defined as there is space between the root surface and the inner boarder of lingual plate. Type B (contact, Fig. 3B) is defined as the root contacts with lingual plate directly and not protrudes into the outer border of lingual plate. Type C (perforation, Fig. 3C) is defined as the root perforates the outer boarder of lingual plate and contacts with lingual soft tissue. Accordingly, in Type C as shown in Supplementary Fig. 1, the thickness of lingual plate was defined as 0 mm and the distance from root apex and the outer surface of lingual plate were also measured. Similarly, we firstly identified the section (Supplementary Fig. 1B) in which the apex was the most distal on the cross-sectional plane by scrolling the image mesially (Supplementary Fig. 1C) and distally (Supplementary Fig. 1D). The lower contact point (D in Supplementary Fig. 1A) between the root apex and outer border of lingual plate was identified and a perpendicular line through point D was made to the horizontal line. Thus the intersection point C was defined and the distance between point C and A was measured. The morphological shapes of lingual plate on these selected cross-sectional images were classified as four subgroups according to the modified classification described by Chan and Momin^27^,^30^ (Type U, undercut on the lingual side; Type P, parallel to the buccal plate; Type S, slanted with buccolingual width reduced on the lingual side, and Type R, round shape on the lingual side) (Fig. 4).

In addition, the impaction depth of these impacted mandibular third molars was evaluated according to the Pell-Gregory classification. As shown in Supplementary Fig. 2, the impaction depth was classified as A (high), B (middle) as well as C (low). Class A was defined when the highest portion of the third molar was above or parallel with the occlusal plane of the second molar, while class B was defined when the highest portion of the third molar is between the occlusal plane and cervical line of the second molar. Class C was classified when the highest portion of the third molar was below the cervical line of the second molar.

### Statistical analyses

All relevant data including the demographic, clinical and radiographic data of patients were collected. Associations between two categorical covariates were assessed by Chi-square test. The quantitative data were compared with Student-*t* test or ANOVA analysis as indicated. The intra-observer agreement of these descriptive CBCT findings was estimated by Cohen κ test. To testify the reliability and reproducibility of our measurement protocol, these measurements in selected patients were performed independently and simultaneously by two trained observers or repeatedly performed by one observer at two time points with one month interval, which were further compared with Student-*t* test. Multiple epidemiological, clinical as well as radiographic parameters were analyzed using logistic regression model to identify the potential risk factors with intimate spatial relationship between root apex of impacted third molars and lingual plate. Statistical analyses were performed using Stata 9.2 software program with indicated methods. *P* < 0.05 (two-sides) was considered statistically significant.

## Additional Information

**How to cite this article**: Wang, D. *et al*. Topographic relationship between root apex of mesially and horizontally impacted mandibular third molar and lingual plate: cross-sectional analysis using CBCT. *Sci. Rep.* **6**, 39268; doi: 10.1038/srep39268 (2016).

**Publisher's note:** Springer Nature remains neutral with regard to jurisdictional claims in published maps and institutional affiliations.

## Supplementary Material

Supplementary Information

## Figures and Tables

**Table 1:** Descriptive data of impacted mandibular third molars.

Variable		No.(%)
Age(years)	30.42 ± 9.71(18–69)	
Gender	Male	117 (52.47%)
	Female	106 (47.53%)
Impaction depth	Class A	99 (27.20%)
	Class B	213 (58.52%)
	Class C	52 (14.29%)
Impaction type	Right side	7 (3.14%)
	Left side	8 (3.59%)
	Bilateral sides	208 (93.27%)

**Table 2:** The distribution of three types of topographic relationship between lingual plate and impacted third molar root on the cross-sectional CBCT view.

	Relationship between lingual plate and impacted third molar root			
	Non-Contact	Contact	Perforation	*P*-value
Age	99	145	120	
18–25	37	68	44	**0.3733**
26–35	39	44	48	
≥36	23	33	28	
Gender				
Male	41	75	79	**0.0013**
Female	58	70	41	
Impaction Depth				
Class A	18	52	29	**<0.0001**
Class B	53	78	82	
Class C	28	15	9	
Number of Roots				
Single root	36	50	23	**0.0067**
Multi-root	63	95	97	
Morphology of Lingual Plate				
Parallel	28	20	11	**0.0010**
Undercut	38	64	73	
Slanted	29	54	31	
Round/Convex	4	7	5	

The numbers in bold indicate statistically significant with *P*-values less than 0.05.

**Table 3:** The thickness of the lingual plate at the level of the root apex of impacted third molar as measured on cross-sectional CBCT image (Point B to C in Fig. 2E).

Type	No. of tooth	Thickness (mm)	*P-*value
Non-contact	99	1.77 ± 0.50	<0.0001^#^
Contact	145	1.35 ± 0.47	
Perforation	120	0.00 ± 0.00*	

*When the tooth apex perforates beyond the outer border of lingual plate as defined as perforation type, the thickness of lingual plate is record as 0 mm. ^#^*P*-value showed here is the result comparing the distance values between non-contact and contact group by Student-*t* test.

**Table 4:** The distance between the root apex of impacted third molar and the outer border of the lingual plate as measured on cross-sectional CBCT image (Point A to C in Fig. 2E and Supplementary Fig. 1D).

Type	No. of tooth	Distance(mm)	*P*-value
Non-contact	99	3.15 ± 0.80	<0.0001^#^
Contact	145	1.35 ± 0.47	
Perforation	120	1.12 ± 0.85*	

*Perforation type is defined when the tooth apex perforates beyond the outer border of the lingual plate. The distance between the root apex and the outer border of lingual plate is measured as shown in Supplementary Fig. 1. ^#^*P*-value showed here is the result comparing the distance values between non-contact and contact group by Student-*t* test.

**Table 5:** Multivariate regression analyses of multiple clinical/radiographic parameters to identify potential risk factors for the topographic relationship between lingual plate and the impacted mandibular third molar root.

Variable		Contact *vs* Non-contact		Perforation *vs* Non-contact	
		*P*	OR (95%CI)	*P*	OR (95%CI)
Age	18–25		1		1
	26–35	0.8869	0.95 (0.48, 1.89)	**0.0457**	2.16 (1.01, 4.59)
	≥36	0.6532	1.20 (0.54, 2.69)	0.2751	1.60 (0.69, 3.72)
Gender	Male		1		1
	Female	0.1273	0.63 (0.34, 1.14)	**0.0274**	0.47 (0.24, 0.92)
Depth of impaction	Class A		1		1
	Class B	**0.0277**	0.47 (0.24, 0.92)	0.8859	0.95 (0.44, 2.04)
	Class C	**0.0001**	0.16 (0.06, 0.39)	**0.0004**	0.14 (0.04, 0.41)
Number of root	Single-root		1		1
	Multi-root	0.3168	0.72 (0.37, 1.38)	0.3229	1.48 (0.68, 3.19)
Morphology of lingual plate	Parallel		1		1
	Undercut	0.0874	1.96 (0.91, 4.22)	**0.0010**	4.45 (1.84, 10.80)
	Slanted	**0.0244**	2.44 (1.12, 5.31)	**0.0374**	2.71 (1.06, 6.92)
	Round	0.2868	2.19 (0.52, 9.32)	0.2809	2.52(0.47, 13.53)

The numbers in bold indicate statistically significant with *P*-values less than 0.05.
