# Supplementary material for: Topographic relationship between root apex of mesially and horizontally impacted mandibular third molar and lingual plate: cross-sectional analysis using CBCT
Source: Sci Rep. 2016 Dec 19;6:39268. doi: 10.1038/srep39268 (PMC5171861; doi:10.1038/srep39268)
Supplement: Supplementary Information [file srep39268-s1.pdf]

Topographic relationship between root apex of mesially and horizontally impacted mandibular third molar and lingual plate: cross-sectional analysis using CBCT

Supplementary Information

Dongmiao Wang[1,2], Xiaotong He[1], Yanling Wang[3], Guangchao Zhou[4], Chao Sun[4], Lianfeng Yang[4], Jianling Bai[5], Jun Gao[2*], Yunong Wu[1*], Jie Cheng[1*]

1. Department of Oral and Maxillofacial Surgery, College of Stomatology
2. Key Laboratory for Neurodegenerative Disease of Jiangsu Province
3. Jiangsu Key Laboratory of Oral Disease
4. Department of Oral and Maxillofacial Radiology, College of Stomatology
5. Department of Biomedical Statistics, College of Public Health

Nanjing Medical University, Nanjing 210029, China

Running Title: Topographic relationship between impacted mandibular third molars and lingual plate

*Address correspondence and reprint requests to:

Prof. Jun Gao, Key Laboratory for Neurodegenerative Disease of Jiangsu Province, Nanjing Medical University, E-mail: **omfs_njmu@163.com**; or **Prof. Yunong Wu**, Department of Oral and Maxillofacial Surgery, College of Stomatology, Nanjing Medical University, E-mail: **yunong_wu@163.com**; or **Prof. Jie Cheng**, Department of Oral and Maxillofacial Surgery, College of Stomatology, Nanjing Medical University, E-mail: **leonardo_cheng@163.com**.

Supplementary Fig.1 Selection of cross-sectional CBCT images when root apex perforates beyond the outer border of lingual plate and measurements

A: The root apex of impacted mandibular third molar which was localized most distally was initially identified on the axial CBCT image. **B-C:** Starting from the image A, the CBCT slice was scrolled distally or mesially. When the root tip was enlarged (**B**) or disappeared (**C**) on the next slices after the image was adjusted mesially or distally (0.1mm) , the present image (**A**) on the cross-sectional plane was further selected for various measurements and spatial analyses. **D:** Schematic illustration of the measurements. Point A was identified as the most lingual site of root apex. A horizontal line through point A was automatically generated. The contact point was identified between root apex and the lower outer border of lingual plate (Point D). A perpendicular line through point D was made to the horizontal line. Then the intersection point C was defined and the distance between point C and A was measured using the digital ruler provided by the software Simplant.

Supplementary Fig.2 The Pell & Gregory classification of impacted mandibular third molars

A-C: Three types of mandibular third molars impaction, class A (**A**), B (**B**),C (**C**), are detected based on the impaction depth similarly as Pell and Gregory classification. The upper white dot line indicates the occlusal plane of the second molar. The lower white dot line indicates the cervical line of the second molar, which parallels with the upper one.

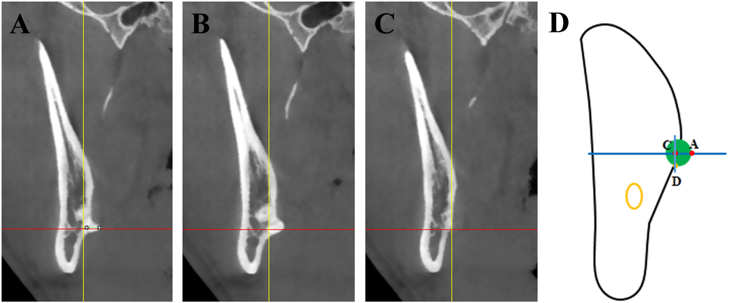
A
B
C
D
C
A
D

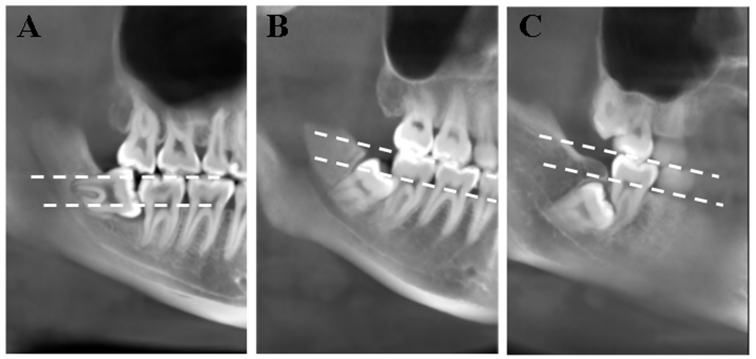
A
B
C

Supplementary Table 1: Difference of the lingual plate thickness at the level of the root apex of impacted third molar as measured independently on cross-sectional CBCT image (Point B to C in Fig.2E) by two observers in randomly selected 30 teeth

Type	No. of tooth	Distance 1# (mm)	Distance 2‡ (mm)	*P*-value
**Non-contact**	11	3.15±0.80	3.05±0.54	0.83
**Contact**	10	1.35±0.47	1.28±0.53	0.79
**Perforation**	9	0.00±0.00*	0.00±0.00*	

Data in Distance 1# was measured by Dr. Dongmiao Wang, while data in Distance 2‡ was determined by Dr. Xiaotong He. They performed these analyses under the same conditions independently. ***When the tooth apex perforates into the lingual plate as defined as perforation type, the thickness of lingual plate is record as 0.00 mm.

Supplementary Table 2: Difference of the lingual plate thickness at the level of the root apex of impacted third molar as measured on cross-sectional CBCT image (Point B to C in Fig.2E) by one observer at two time points (one month interval) in randomly selected 30 teeth

Type	No. of tooth	Distance 1# (mm)	Distance 3¶ (mm)	*P*-value
**Non-contact**	11	3.15±0.80	2.98±0.62	0.66
**Contact**	10	1.35±0.47	1.30±0.59	0.72
**Perforation**	9	0.00±0.00*	0.00±0.00*	

Both data in Distance 1# and 3¶was measured by Dr. Dongmiao Wang. He performed these analyses under the same conditions independently and at two time points with one month interval. ***When the tooth apex perforates into the lingual plate as defined as perforation type, the thickness of lingual plate is record as 0.00 mm.
